# Hydrogen: A Rising Star in Gas Medicine as a Mitochondria-Targeting Nutrient via Activating Keap1-Nrf2 Antioxidant System

**DOI:** 10.3390/antiox12122062

**Published:** 2023-11-30

**Authors:** Danyu Cheng, Jiangang Long, Lin Zhao, Jiankang Liu

**Affiliations:** 1 Center for Mitochondrial Biology and Medicine, The Key Laboratory of Biomedical Information Engineering of Ministry of Education, School of Life Science and Technology, Xi’an Jiaotong University, Xi’an 710049, China; dycheng0403@stu.xjtu.edu.cn (D.C.); jglong@mail.xjtu.edu.cn (J.L.); 2 Cardiometabolic Innovation Center, Ministry of Education, Department of Cardiology, First Affiliated Hospital of Xi’an Jiaotong University, Xi’an 710061, China; 3 School of Health and Life Sciences, University of Health and Rehabilitation Sciences, Qingdao 266071, China

**Keywords:** hydrogen, gas medicine, antioxidant, mitochondria, Keap1-Nrf2, Nrf2 activator

## Abstract

The gas molecules O_2_, NO, H_2_S, CO, and CH_4_, have been increasingly used for medical purposes. Other than these gas molecules, H_2_ is the smallest diatomic molecule in nature and has become a rising star in gas medicine in the past few decades. As a non-toxic and easily accessible gas, H_2_ has shown preventive and therapeutic effects on various diseases of the respiratory, cardiovascular, central nervous system, and other systems, but the mechanisms are still unclear and even controversial, especially the mechanism of H_2_ as a selective radical scavenger. Mitochondria are the main organelles regulating energy metabolism in living organisms as well as the main organelle of reactive oxygen species’ generation and targeting. We propose that the protective role of H_2_ may be mainly dependent on its unique ability to penetrate every aspect of cells to regulate mitochondrial homeostasis by activating the Keap1-Nrf2 phase II antioxidant system rather than its direct free radical scavenging activity. In this review, we summarize the protective effects and focus on the mechanism of H_2_ as a mitochondria-targeting nutrient by activating the Keap1-Nrf2 system in different disease models. In addition, we wish to provide a more rational theoretical support for the medical applications of hydrogen.

## 1. Introduction

**Figure 1:**
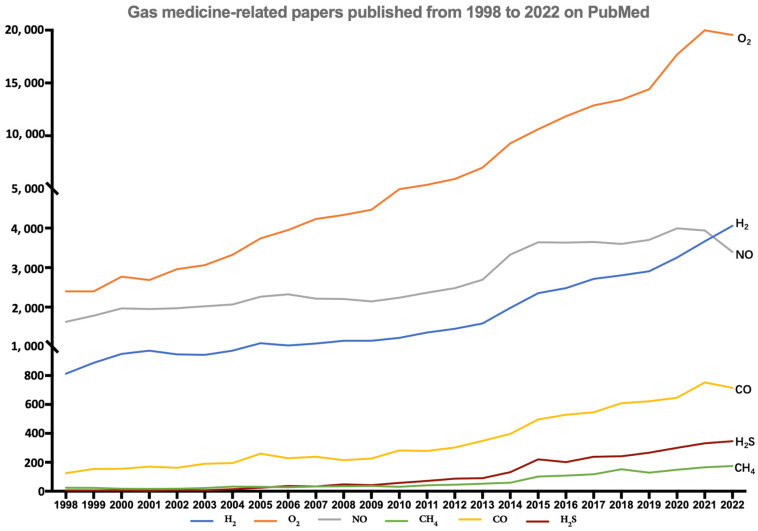
The number of papers published from 1998 to 2022 about the most widely used gases (H_2_, CO, NO, O2, CH_4_, H_2_S) in medicine (data obtained after a keyword index search on PubMed with “gas name + medicine”).

Gas molecules are increasingly being used for medical purposes and their application has developed into a separate field of medicine. The gases most widely used in medicine include oxygen (O_2_), nitric oxide (NO), methane (CH_4_), carbon monoxide (CO), hydrogen sulfide (H_2_S), and hydrogen (H_2_). As shown in Figure 1, the number of articles related to medical gas molecules has grown substantially from 1998 to 2022, especially articles related to O_2_ and H_2_.

O_2_ and NO are the two medical gas molecules that most attract researchers’ attention, with tens of thousands of studies focusing on these two gases published since as early as the 1990s. O_2_ is the most crucial gas for all living organisms on earth and accounts for around 1/5 of the volume of air. As an important gas to maintain human respiration, O_2_ is mainly used to provide supplemental respiration for the sick, astronauts traveling in space, mountaineers, etc. In addition, it has the function of destroying bacteria. Due to the importance of O_2_, the 2019 Nobel Prize in Physiology or Medicine was awarded to William G. Kaelin Jr., Sir Peter J. Ratcliffe, and Gregg L. Semenza, who discovered how cells sense and adapt to the availability of O_2_ [1]. NO, commonly applied in the biomedical field in the form of NO· as a free radical gas, was found to work as a mediator of cell-to-cell communication in vasodilatation, inflammation, and neurotransmission at the end of the last century. Robert F. Furchgott, Louis J. Ignarro, and Ferid Murad et al. demonstrated that NO· is an important signaling molecule in the cardiovascular system, and this discovery won the 1998 Nobel Prize in Physics or Medicine [2].

CH_4_ is the simplest of the organic compounds. For decades, CH_4_ was thought to have almost no physiological role, while in the last few years, scientists have realized that CH_4_ can play important biological roles such as anti-inflammatory, antioxidant, and antiapoptotic roles. As a result, CH_4_ has been used as a gastric decontaminant in emergency clinical settings of poisoning or drug overdose and serves as a passive indicator of colonic function [3].

CO and H_2_S have long been known as hazardous factors. Long-term exposure to environments which are rich in CO may be fatal. However, a growing amount of research suggests that CO is an important gaseous mediator along with NO· and H_2_S. Endogenously produced or inhaled CO has important physiological functions in regulating vascular function, inflammation, apoptosis, cell proliferation, and signaling pathways. Studies have shown that inhaled CO suppresses chronic inflammation in patients with stable chronic obstructive pulmonary disease (COPD) [4]. Initially, H_2_S was regarded as a harmful gas since exposure to H_2_S irritates the eyes and respiratory system. However, scientists have now shown that H_2_S is an essential physiological factor, as it is produced by bacteria in the human oral cavity and gastrointestinal tract. As the least appreciated of the three gaseous mediators (gas transport mediators), it is now considered to be an important gas transport mediator after NO· and CO. H_2_S has been shown to modulate many physiological processes such as vasodilation, anti-inflammation, resistance to oxidative stress, protection against ischemia-reperfusion injury, etc. [5].

As the smallest of all molecules, the functions of H_2_ have also caught the eye of scientists in the field of biomedicine. As early as the beginning of the last century, H_2_ was first tested as a diving gas, proving that it is the best breathing medium for medium and deep diving and is safe for the organism, with no toxic side effects found. To date, H_2_ biomedicine has investigated the effects and mechanisms by which H_2_ molecules, including H^+^ ions (pH) and H^−^ ions (a powerful reducing agent existing as MgH_2_, CaH_2_, AlLiH_4_, etc.) and heavy H_2_ (deuterium and tritium), act in various diseases [6].

## 2. History of H_2_ Medicine

H_2_ is known to be a colorless, odorless, and tasteless gas that is chemically stable [7]. In general, around 35 mL to 321 mL of H_2_ is produced and released through bacterial fermentation by the human digestive system per day [8]. Several ways are used to ingest or consume H_2_, such as drinking or injecting H_2_ water (HW), inhalation of H_2_, bathing in HW, dropping H_2_ saline into the eyes, etc. H_2_ plays an anti-inflammatory and antiapoptotic role through its selective antioxidant properties and has become a unique cytoprotective agent [9].

H_2_ used to be considered an inert gas not involved in any life activity. It was not until 1975 that Dole et al. found significant regression of mouse skin tumors in squamous cell carcinoma mice exposed for a fortnight to a mixture of 97.5% H_2_ and 2.5% O_2_ at a total pressure of eight atmospheres, first confirming the medical usefulness of H_2_ [10]. Unfortunately, this study has not attracted academic attention due to the technical difficulties of applying hyperbaric H_2_ therapy in a clinical application.

In 1996, Chinese scientist Yuanwei Du noticed the significance of H_2_ for life [11]. Dr. Du believes that excessive accumulation of peroxides produced in the metabolic process is the root cause of various diseases and aging; the organism must have a certain mechanism to fight against these peroxides. H_2_ is a reducing agent which can eliminate peroxides naturally without side effects, making creatures achieve a balance in the sense of redox balance. In Du’s experiment, tritium gas was produced by electrolysis of tritium water. The tritium gas was then fed, instead of H_2_, into the mouse’s living environment. He found that tritium was present in all tissues and organs of mice, which means that tritium gas is involved in the life activities of living organisms by transforming into tritium ions prevalent in living organisms, indirectly proving that the H_2_ in air is both a constituent substance and an energetic substance of life. This experiment also proves the basic mechanism of H_2_ metabolism. A number of H_2_ medicine-related papers published by Yuanwei Du at the end of the 20th century further confirmed that H_2_ produced by water electrolysis has a pronounced effect on the vital activities of plants (lilac branches), animals (mice), as well as humans [12]. Du’s work creatively combines the physiological effects of H_2_ with the free radical aging theory, explains the antioxidant activity of H_2_ molecules, and confirms that H_2_ may have an immeasurable effect on a wide range of diseases.

In 2007, Ohsawa et al. from the Nippon Medical School published an important article on H_2_ medicine in the journal *Nature Medicine* [13]. This study used a low concentration of H_2_ (1–4%) for inhalation over a short period (35 min) by mice and found positive effects in the treatment of cerebral ischemia-reperfusion injury, showing that short-term inhalation of a low concentration of H_2_ for the treatment of the disease is feasible. They proposed a mechanism whereby H_2_ could act as a therapeutic antioxidant, selectively reducing cytotoxic oxygen radicals (^•^OH and ONOO^−^), leading to the inhibition of cerebral ischemia-reperfusion injury. Because this study was published in the prestigious *Nature Medicine* journal, it provided a broad prospect for both basic and clinical research on H_2_ and brought H_2_ medicine to the attention of a wide range of academic cycles. Since then, more and more scholars have joined the research on H_2_ medicine to explore its effects on various diseases such as inflammation, drug toxicity, and obesity. More than a thousand peer-reviewed research papers have been published to date.

In the beginning, scientists focused mainly on acute and chronic organ injuries related to oxidative stress, such as those found in animal experiments investigating drug toxic injury or ischemia-reperfusion injury in vital organs such as the heart and liver. During this period, researchers mostly used diverse injury models to validate the therapeutical effects of H_2_ inhalation. Between 2009 and 2012, more research began to appear on drinking H_2_-enriched water (HRW) [14], injecting H_2_-enriched saline (HRS) [15], as well as studies on boosting H_2_ replenishment through gut bacteria [16]. Meanwhile, a number of clinical studies have used HRW in the treatment of diseases including metabolic syndrome, Parkinson’s disease, hemodialysis, sports injuries, and rheumatoid arthritis [9]. For the past few years, on the foundation of previous studies, H_2_ medicine research has studied molecular mechanisms, especially focusing on the molecular pathways of inflammation and oxidative stress mediated by H_2_. However, regarding the molecular mechanism of H_2_, most scholars have followed the view of Ohsawa et al. in their paper in *Nature Medicine*: that H_2_ is a selective hydroxyl radical (^•^OH) scavenger. As a result, most scholars have focused on the antioxidant mechanisms of H_2_ based on this classification [17,18]. Nevertheless, some scholars have proposed that H_2_ plays a signaling role that may be involved in metabolic processes and may even provide energy for bacteria, which broadens thoughts on the development of H_2_ research [19,20,21].

A number of Chinese researchers have devoted themselves to developing H_2_ medicine. These researchers has received more than 80 grants from the National Natural Science Foundation of China and have published hundreds of basic and clinical academic papers. Prof. Xuejun Sun of the Second Military Medical University is one of the leading figures in H_2_ medicine in China. Prof. Sun’s group engaged in the diving hyperbaric medicine research for a long time. The most important research object of diving hyperbaric medicine is the types of gases that can be breathed by human beings, with H_2_ being one of the key types of gases in the field of diving hyperbaric medicine. Sun’s group focuses on the biological effects of H_2_ and its application in medicine for the first time, revealing the value of H_2_ in medicine in China. Moreover, Prof. Sun participated in organizing several international symposiums on H_2_ medicine, inviting experts from all over the world to discuss the future of H_2_ medicine. His team collaborates with medical organizations around the world to carry out research on the application of H_2_ medicine and to expand the scope of H_2_ applications in the medical field.

Prof. Shucun Qin of Shandong First Medical University is another key promoter of H_2_ molecular medicine in China. Prof. Qin established the first H_2_ Biomedical Research Institute at the university in 2015, training a number of key researchers in H_2_ medicine. He established the standardized laboratory for H_2_ molecular biology that has published multiple placebo-controlled population trials, providing important clinical evidence for the translation of H_2_ into medicine. Qin’s recent review summarizes 51 clinical trials involving 1213 subjects in four areas of H_2_ biomedicine: basic research, exercise, dermatology, and healthcare [22]. The results showed that H_2_ can reduce oxidative stress damage caused by strenuous exercise, reduce lactic acid build-up after exercise, prevent exercise acidosis, and reduce exercise fatigue. In addition, H_2_ intervention can play a positive role in skin beauty and improve cardiovascular health.

Prof. Xuemei Ma’s team at the Beijing Institute of Technology was also an early group of H_2_ medicine researchers in China. Prof. Ma is committed to elucidating the biological basis of H_2_ medicine at the molecular, cellular, and holistic levels, conducting in-depth basic research and clinical translational research, especially on the mechanism of H_2_ molecules on tumor prevention. Her team has verified that H_2_ can inhibit the proliferation of gliomas (Gliomas) by inducing glial stem cell differentiation in in vitro and in vivo experiments [23].

In addition to these key researchers, there are hundreds of scientists doing work on H_2_ medicine, including the Chinese academicians Prof. Nanshan Zhong, Zhaofen Xia, Hongyang Wang, and young scientists like Prof. Qianjun, who proposed the concept of H_2_ nanomedicine to address the issues of H_2_ medicine by using functional micro/nanomaterials for augmented H_2_ therapy in cancer, and Wenbiao Shen, who is devoted to the application of H_2_ in agriculture. An academic association for H_2_ medicine with more than 400 members has been formed. As of today, current clinical studies on H_2_ are still continuously emerging, and the scale of the studies is gradually expanding. With its favorable biosafety and convenience, an H_2_ inhalation device has been included in the Chinese National Medical Products Administration’s new medical device development process (Registration Approval No. 20203080066). Moreover, in Japan, H_2_ has been approved as a food supplement [9].

## 3. H_2_: A Mitochondria-Targeting Molecule/Nutrient Rather Than a Selective ^•^OH Scavenger

Sustained oxidative stress leads to the onset and progression of many common diseases. To date, little has been achieved in this regard, although a large number of studies have attempted to develop an effective antioxidant without side effects. Mitochondria, as a major source of oxidative stress, is considered a new therapeutic target for small molecule interventions [24]. H_2_ suppresses reactive oxygen species (ROS) accumulation, inhibits the cell death program, and maintains mitochondrial structure and function [25,26]. Preliminary clinical trials suggest that drinking H_2_ dissolved in water appears to improve the pathology of mitochondrial disease [27,28].

Mitochondria have a double membrane structure that forms the difference in potentials between the inner and outer membranes and controls the movement of diverse molecules and factors (e.g., ions) in and out of the organelle while affecting mitochondrial stability. Although the outer membrane is comparatively permeable to small molecules and large proteins (which are transported by diffusion or transposases), the inner mitochondrial membrane is highly impermeable to most molecules [29]. Special membrane transport proteins (e.g., TIM-TOM (preprotein translocase of the inner membrane of mitochondria-preprotein translocase of the outer membrane of mitochondria) complex, etc.) are needed for all ions and molecules to enter or leave the mitochondrial matrix. This means most antioxidants cannot enter the mitochondria to effectively scavenge ^•^OH [30,31]. The difference with other antioxidants is that, as the smallest molecule in nature, H_2_ can easily spread and penetrate into the cell membrane to react with organelles such as mitochondria and the nucleus [32].

While the idea that H_2_ is a selective antioxidant has been popularized [7], it is still not known whether the effects of H_2_ arise from the direct reaction with ^•^OH or from the inhibition of ^•^OH production. Let us first provide some basic information on free radicals.

As we know, ^•^OH is generated by the Haber−Weiss reaction:

O_2_^•−^ + H_2_O_2_ → O_2_ + ^•^OH + OH^−^

This reaction is thermodynamically feasible but kinetically too slow. So, ^•^OH is mainly generated by the Fenton reaction:

Fe^2+^ + H_2_O_2_ → Fe^3+^ + ^•^OH + OH^−^

The three main properties of ^•^OH are below: 1. Short life: ^•^OH has a very short half-life (10^−9^ s, or 1 ns, whereas the half-life of superoxide is 15 s) and requires no time to diffuse (no more than 50 molecular diameters from the site of formation), so the reaction is local with the antioxidant found where ^•^OH is produced. 2. High reactivity: ^•^OH is the ROS with the highest reduction potential; compared to other oxygen species, it reacts with extremely high rate constants (high reactivity) that approach diffusion-limited rate constants, with rate constants of 10^9^–10^10^ M^−1^ s^−1^. So, ^•^OH is the strongest (most powerful) oxidant of the oxyradicals. 3. Unselective and indiscriminate: ^•^OH reacts unselectively and indiscriminately with almost every type of molecule found in living cells, including lipids, proteins, amino acids, DNA, RNA, and sugars. Therefore, the best antioxidant is not a ^•^OH scavenger but, rather, an iron chelate to prevent the generation of ^•^OH.

The reaction with many substances in the body occurs at a rate that exceeds that of H_2_, which means that H_2_ has difficulty competing with these molecules effectively in the body, especially when H_2_ is at a relatively lower concentration than other endogenous substances. Biokinetic analyses of the intracellular reactions of ^•^OH/ONOO^−^ show that intracellular molecules, such as nucleic acids and amino acids, react with ^•^OH more readily at a significantly faster rate than H_2_ [33,34], which implies that H_2_ can hardly act as an ^•^OH scavenger or barely directly react with ^•^OH.

In 2005, we first proposed the new concept of “mitochondrial nutrient”. The so-called “Mitochondrial nutrients” refer to any compound that can protect mitochondria from damage, repair mitochondria injury, and promote mitochondrial function. Their mechanisms of action may include (1) protecting mitochondrial enzymes and/or stimulating enzyme activity by increasing the levels of substrate and cofactors; (2) inducing the activation of endogenous antioxidant systems, such as phase II enzymes, to enhance antioxidant defense; (3) preventing mitochondria from producing ROS and removing ROS in mitochondria, and (4) protecting and repairing mitochondrial damage, including energy promoters [35,36,37].

Researchers in our lab reported that in an LPS-induced lung injury mouse model, hyperoxic HRS effectively reduced mitochondrial swelling and cristae breaks and significantly reversed the reduction of mitochondrial complex I, IV, and V activities [38,39]. Not coincidentally, in a high-fat diet (HFD)-induced liver injury model, coral calcium hydride (CCH, a solid form of molecular H_2_ carrier made from coral calcium) treatment improved glucose and lipid metabolism, ameliorated hepatic mitochondria abnormalities, restored the protein expression and the activity of complex II, and activated phase II enzymes [38,39]. These studies imply that H_2_, as a highly promising mitochondrial nutrient, is able to target mitochondria.

Ohsawa et al. [13] used antimycin A (an inhibitor of mitochondrial respiratory complex III) to induce excess O_2_^•−^ production. In this model, O_2_^•−^ rapidly converted to H_2_O_2_, which was further converted to ^•^OH. Their result showed that H_2_ treatment prevented the decrease in mitochondrial membrane potential caused by antimycin A treatment. They concluded that H_2_ protects mitochondria from ^•^OH damage. The researchers hypothesized that H_2_ enters the mitochondria and acts on the mitochondrial respiratory chain, weakening the Fenton reaction by inhibiting transition metal activity and, ultimately, inhibiting ^•^OH production but not scavenging ^•^OH directly [40]. Lebaron et al. suggested that H_2_ exerts a hormetic-like effect as a redox adaptogen because it can exhibit pro-oxidative activities while also reducing excess oxidative stress [41].

Accordingly, H_2_ is considered as a potential and promising mitochondria-targeting molecule or nutrient that acts as a redox homeostasis regulator [42].

**Figure 2:**
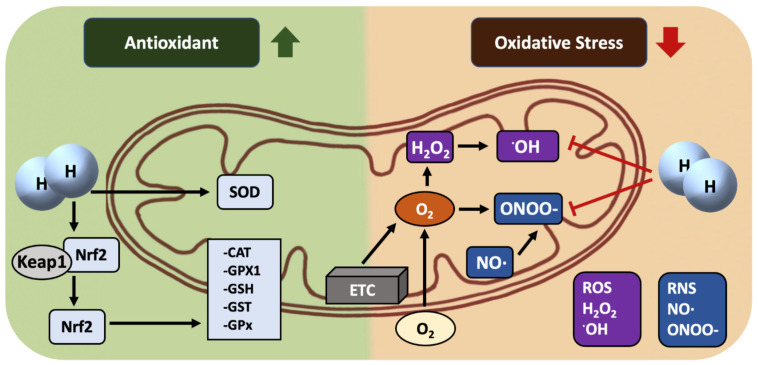
Mechanisms of mitochondria-targeting by molecular H_2_: (1) barely reacting with ^•^OH and ONOO^−^ directly; (2) mainly activating Keap1-Nrf2 antioxidant systems indirectly (SOD: superoxide dismutase, CAT: catalase, GPX1: glutathione peroxidase 1, GSH: glutathione, GST: glutathione S-transferase, GPx: glutathione peroxidase, ETC: electron transfer chain, ^•^OH: hydroxyl radical, ONOO^−^: nitrite peroxide, NO: nitric oxide radical).

As is well known, H_2_ is a moderate/mild reducing agent (the standard reduction potential of H^+^/H_2_ at PH7 is −0.42, stronger than NAD^+^/NADH (−0.32) but weaker than acetate/acetaldehyde (−0.60)), barely able to scavenge ^•^OH directly in a living body (Figure 2). Because mitochondria are the main sites of ROS generation and the targets of ROS, we suggest that the more important mechanism of the H_2_ molecule may be that it can easily enter cells and subcellular organelles, including mitochondria, to play a protective role through its strong penetration ability, subsequently activating the Keap1-Nrf2 (Kelch-1ike ECH-associated protein l, nuclear factor erythroid 2-related factor 2) antioxidant defense system to inhibit oxidative damage and improve the mitochondrial function, and, finally, improving the prevention of various diseases. H_2_ has been shown to significantly activate the Keap1-Nrf2 system, regulate the activities of endogenous antioxidants, and enhance the ability of cells to fight against damage [43].

## 4. The Mechanisms of H_2_ as an Nrf2 Activator

Nrf2 is a key factor in the regulation of oxidative stress which belongs to the CNC-BZIP transcription factor family. Upon normal physiological conditions, Nrf2 binds to Keap1 to form a complex present in the cytoplasm in a low-activity state [44]. When the organism is stimulated by oxidative stress or other pathological conditions, the cysteine residue of Keap1 is modified or Nrf2 is phosphorylated; then, Nrf2 is released from the complex and translocated to the nucleus, where it binds to the antioxidant response elements (AREs) sequence in the nucleus, initiating NRF2-mediated transcriptional processes to activate a series of phase II antioxidant enzymes to generate antioxidants to scavenge ROS and other harmful substances.

It is reported that Nrf2 can be activated in various ways, among which the Keap1-Nrf2 pathway is the most classical Nrf2 activation pathway. Keap1 contains multiple oxidative stress response sensor proteins which have different physiological functions in response to different forms of stress. To date, several studies have demonstrated that H_2_ activated Nrf2 through the Keap1-Nrf2 system [45,46], but the clear mechanism of the activation is not known.

Nrf2 inducers are diverse; most are electrophilic and readily react with Keap1 through the cysteine thiol groups. Among them, Cys151/Cys273/Cys288 plays a fundamental role in the perception of electrophilic Nrf2-inducing chemicals. Therefore, Nrf2 inducers have been divided into different categories based on the different cysteine residues of Keap1 they react with (Table 1). The first class specifically targets the Cys151 sensor, such as medically relevant bardoxolone methyl. Bardoxolone methyl acts as an electrophilic inducer of Nrf2 that forms a covalent interaction with the Cys151 residue of Keap1, thereby inhibiting Nrf2 ubiquitination. In mice, the Cys151 point mutation in Keap1 eliminated Nrf2 signaling and the hepatoprotective effect of bardoxolone methyl in vivo [47]. The second class of inducer targets Cys288, and 15-deoxy-prostaglandin J2 (15d-PGJ2) has been identified in this group. 15d-PGJ2, one of the endogenous Nrf2 inducers synthesized from arachidonic acid, forms a covalent compound with Keap1 to compete for the Keap1-Nrf2 binding. Class III inducers, such as 4-hydroxynonenal (4-HNE), target Cys151/Cys273/Cys288. Mass spectrometry analysis revealed that 4-HNE directly modifies cysteine residues on Keap1 and deregulates its inhibition of Nrf2 by inhibiting Keap1, further increasing the expression levels of Nrf2 target genes (e.g., TXNRD1, thioredoxin reductase-1) [48]. Indeed, Nrf2 activation was significantly reduced when Cys151 was mutated, whereas Nrf2-induced target gene activation was only slightly affected when Cys273 and Cys288 residues were mutated [49,50].

In addition, we classify the electrophilic compound that activated Nrf2 on the cysteine residues other than Cys151/Cys273/Cys288 as Class IV. The compounds of this group include, for example, Pubescenoside A, which acts on Cys77/Cys434.

Moreover, several inducers activate Nrf2 in a more complex way than the previously identified electrophilic sensors that bind to Cys226, Cys613, Cys622, and Cys624. We classify them as Class V. Hydrogen peroxide (H_2_O_2_), a key ROS molecule important in cellular physiology, is representative of this classification. Suzuki et al. revealed that Keap1 uses cysteine residues to create a special mechanism to make a disulfide bond between any combination of Cys226, Cys613, Cys622, and Cys624 to sense H_2_O_2_ [51]. This sensing mechanism is different from that used by the electrophilic Nrf2 inducer.

There is also a type of inducer that does not act through the cysteine of Keap1; these have been classified as a Class VI, and they directly inhibit the interaction between Keap1 and Nrf2 and include non-electrophilic protein–protein interaction inhibitors (PPIs) [52]. Horie et al. suggested that Keap1 binding to Nrf2 is a “hinge and latch model”, with PPIs actively using a hinge-locking mechanism, whereas electrophilic Nrf2 activators do not use this mechanism when activating Nrf2 [53].

**Figure 3:**
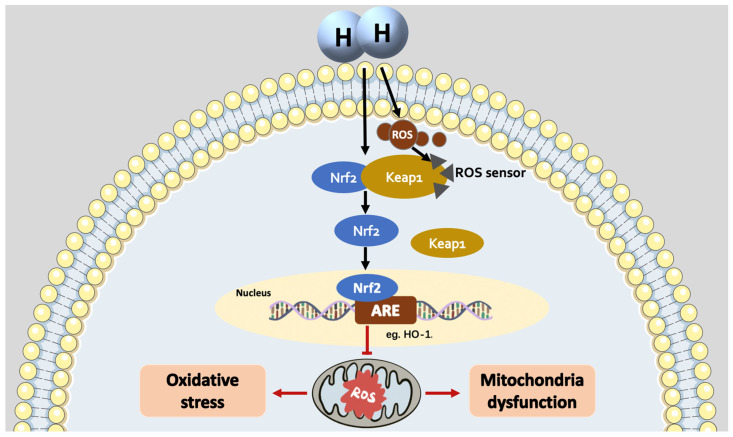
H_2_ may activate Nrf2 and its mediated phase II enzyme system via non-electrophilic protein–protein interactions or by inducing the production of excess ROS to oxidized intracellular Keap1 and then activating Nrf2.

The mechanism of Nrf2 activation by H_2_ seems different from the mechanism of perception of electrophilic Nrf2 inducers but may be closer to the mechanism of Class V and VI (Figure 3). As the smallest and one of the simplest molecules, H_2_ molecules have the capacity to pass through the Keap1 and Nrf2 binding structure to inhibit the interaction between Keap1 and Nrf2, playing the role of Class VI activators [51]. It has also been suggested that the mechanism by which H2 activates Nrf2 may be similar to that of H_2_O_2_ (Class V), either by promoting mitochondrial respiratory activity, resulting in inducing excess ROS, or by opening the mitochondrial-(ATP) K^+^ channel to generate ROS, which then oxidizes intracellular Keap1, releasing Nrf2 [54].

Notably, recent studies have pointed out that the oxidized form of iron porphyrin bound to the OH group is considered to be a redox-related biosensor for H_2_, buffering the high oxidative electrophilicity of ^•^OH. When the originally oxidizing and deleterious electrophilic properties of ^•^OH are mitigated, the resulting electrophilic potency may activate Nrf2, with an effect similar to that of the hormone. However, this viewpoint is brand new and still needs further verification [55].

To date, the activation of Nrf2 and its mediated antioxidant enzyme system by H_2_ has been reported in a variety of tissue-associated diseases, including brain, lung, liver, heart, ovary, and kidney diseases [45,56,57]. In Nrf2-deficient mice, the ability of H_2_ against oxidative stress in the lung was significantly diminished [58].

The results of studies of neuroblastoma cells showed that exposure of SH-SY5Y cells to H_2_ increased the production of mitochondrial superoxide. This process was accompanied by Nrf2 nucleus translocation as well as increased expression of Nrf2-regulated antioxidant enzymes, suggesting that H_2_ alleviates mitochondrial oxidative stress through activating Nrf2 [59]. Inhaled H_2_ also reduces neuroinflammation in memory-related regions through increasing Nrf2 protein expression in a sepsis-induced blood–brain barrier impairment and memory dysfunction [60,61]. Interestingly, one of the studies we were involved in reported that H_2_ (2–4%) protected against delayed encephalopathy after acute carbon monoxide poisoning, and this protective effect was related to the involvement of Nrf2 and its mediated phase II enzyme system [62].

Similar results were obtained in the lung from a seawater instillation-induced acute lung injury rabbit or from cecal ligation and puncture-induced sepsis mice, which proved that H_2_ could regulate the expression of heme oxygenase-1 (HO-1), the Nrf2 downstream antioxidant protein [63,64]. Inhaled H_2_ significantly alleviated the drop in blood O_2_ during hyperoxic exposure, remitted lung inflammation, and upregulated HO-1 expression. In a sepsis-induced acute lung injury model, H_2_ molecules inhibited high-mobility group protein1 (HMGB1) expression by activating the Nrf2-HO-1 pathway [65,66]. The latest research has revealed that H_2_ also affected COVID-19-induced lung injury via Nrf2 [67].

Sun et al. [46] demonstrated that the administration of H_2_ reduced oxidative stress in LPS-treated mice livers through activation of the Keap1-Nrf2 system. Moreover, Liu et al. [56] reported that H_2_ improved lipid accumulation by modulating the miR-136/MEG3/Nrf2 pathway in non-alcoholic fatty liver disease.

In an ischemia model induced in the H9C2 cell line, a H_2_ gas-rich medium reduced the production of ^•^OH, promoted Nrf2 nuclear translocation, and regulated the Nrf2-HO-1 pathway, suggesting that H_2_ can preserve ischemic cardiomyocytes by stimulating the Nrf2 pathway [68]. H_2_ ameliorated LPS-injured HUVECs and inflammatory responses through Nrf2 and its downstream protein HO-1 [69].

In a long-term cyclosporine A (CsA)-induced nephrotoxicity model, HRW reduced ROS and MDA levels, increased the activities of GSH and SOD, and then improved the vascular and renal functions of rats with renal damage. Meanwhile, HRW significantly decreased the level of Keap1 while increasing the expression of Nrf2, NADPH dehydrogenase quinone1, and HO-1, suggesting that HRW restored the balance of the redox state and improved CsA-induced renal function by activating the Keap1-Nrf2 signaling pathway [45].

In a rat model with ovarian injury induced by cisplatin, HRS recovered the activity of SOD and catalase, reduced MDA levels in serum and ovarian tissues, as well as increased ovarian Nrf2 expression [70]. Inhalation of 2% H_2_ also attenuated severe sepsis-induced intestinal injury by modulating HO-1 and HMGB1 release in mice [71].

## 5. The Medical Effects of H_2_: Focus on the Effect on Mitochondria

A great number of basic and clinical studies have found that H_2_ is an important physiological regulator that protects against tissue-related diseases, such as those of the lung, heart, central nervous system, kidney, pancreas, etc., through protective effects such as antioxidant, anti-inflammatory, and antiapoptotic effects. Mitochondrial dysfunction is closely related to disease development [36]. In this section, we focus on the effects of H_2_ on mitochondrial function in different diseases.

### 5.1. Effects of H_2_ on Respiratory System Diseases

To date, molecular H_2_ has been reported to have positive effects in the prevention and treatment of acute lung injury, chronic obstructive pulmonary disease, asthma, and pulmonary hypertension [67]. Of interest, the National Health Commission of China (NHC 7th Edition Trial: Beijing, 2020) and the Chinese Centre for Disease Control and Prevention (CDCP 6th Edition Trial: Beijing, 2020) recommend effective O_2_ therapy as one of the modalities for the general treatment of patients with COVID-19. They also noted that inhalation of a mixture of molecular H_2_ and O_2_ (66.6% H_2_ & 33.3% O_2_) is more effective than inhalation of O_2_ alone [72]. The research in our lab showed that H_2_ enriched and that oxygenated saline inhibited LPS-induced lung injury in C57BL/6 mice through the NF-κB/NLRP3 signaling pathway. H_2_ demonstrated a more significant effect in inflammatory and antiapoptotic mechanisms, while O_2_ enhanced the hypoxic effect of the organism, with the combined protective effect of the two gases being better than their respective effects [39].

Inhalation of 2% H_2_ improves mitochondria function through increased mitochondrial-membrane potential and ATP levels and promotes the activity of mitochondrial-respiration complex I and complex II. H_2_ also regulates mitochondria dynamics, which decreases the expression of mitochondria fission protein Drp1 but increases the expression of mitochondria fusion protein mitofusin-2 (MFN2) [73].

Post-transplant morbidities, such as graft ischemia-reperfusion damage and graft-versus-host disease, are key challenges in transplantation. H_2_ acted as a prophylactic agent against post-transplant complications in several animal models of organ transplantation [74]. In a rat lung transplantation model, the combination of mechanical ventilation and prolonged cold ischemia resulted in a significant reduction of gas exchange in rat lung tissue (treatment with 98% O_2_ plus 2% nitrogen), while treatment with 98% O_2_ plus 2% H_2_ inhibited the increased tendency of pro-inflammatory cytokines and apoptotic molecules and upregulated the expression of HO-1 in the lung grafts [75]. Not only that, H_2_ molecules inhibited the levels of proapoptotic proteins caspase-3 and caspase-8 in lung grafts, activated the expression of antiapoptotic proteins Bcl-2 and Bcl-xL, and stabilized the mitochondrial outer membrane, preventing the release of cytochrome c into the cytosol [76]. In addition, advanced treatment of rat lung donors with H_2_ induces the gene expression of stress response and ATP synthesis [77].

H_2_ is considered to be a potential radioprotective agent [78]. In radiation-injured lung epithelial cell line A549, H_2_ downregulates the gene expression of proapoptotic Bax and inhibits its translocation to mitochondria through an unknown mechanism [79].

### 5.2. Effects of H_2_ on Cardiovascular System Diseases

Molecular H_2_ has shown many benefits in cardiovascular disease (CVD) applications and can be used to treat a wide range of CVDs that cover ischemia-reperfusion injury, atherosclerosis, cardiac hypertrophy, radiation-induced cardiac damage, and chemotherapy-induced cardiotoxicity [80,81,82]. We evaluated the influence of inhaled H_2_ on heart and nerve function after cardiopulmonary resuscitation by comparing the effects of H_2_ inhalation in a rat model of cardiac arrest asphyxiation. The results showed that compared with O_2_, serum troponin T and S100B were significantly reduced after inhaling H_2_. In the meantime, left ventricular ejection fraction, cardiac function, and neurological function were significantly improved after H_2_ inhalation [82].

H_2_ increases autophagy by promoting autophagic flow, thereby alleviating harmful stress [83]. HRS was found to promote PINK1/Parkin-mediated autophagy, activate mitochondrial autophagy, cause damaged mitochondria to be engaged by lysosomes, and further ameliorate the inflammatory response and apoptosis induced by myocardial ischemia/reperfusion (MI/R) [84]. Feng et al. reported that HRS combined with early aerobic exercise enhances acute myocardial infarction-induced superoxide dismutase levels and total antioxidant capacity, promotes mitochondrial and DNA repair by partially regulating the expression of antioxidant-associated proteins and mitochondria-associated proteins, and protects against myocardial injury after MI [85].

HRW protects cardiac and aortic graft recipients from inflammation-related deterioration and improves allograft survival by decreasing endothelial cell proliferation, inhibiting T-cell proliferation, and reducing oxidative stress in a heterotopic heart transplantation rat model [86]. This protection mechanism also correlates with ATP levels and increases the enzyme activity of complex II, III, and V on the mitochondrial respiratory chain.

Sepsis is associated with systemic infections and inflammatory responses induced by the cardiovascular system [87]. In a sepsis-induced, myocardial-injured mouse model, molecules H_2_ promoted protein increase of HO-1, MFN2, and PGC1-1α expression, inhibited sepsis-induced mitochondrial dysfunction, and remodeled fatty acid oxidation in the heart in the sepsis model by increasing myocardial energy [88,89].

Oxidative stress is a major risk factor for worsening LV hypertrophy. Yu et al. found that H_2_ saline water improves mitochondria function by restoring electron transport chain enzyme activity, inhibiting ROS formation, and increasing ATP production in spontaneously hypertensive rats with LV hypertrophy. H_2_ saline water also inhibits oxidative stress, inflammatory processes, and angiotensin II [90].

Zhang et al. found that HRS treatment ameliorates vascular functional abnormalities, such as aortic hypertrophy and endothelial dysfunction, in spontaneously hypertensive rats by alleviating oxidative stress, restoring pressure receptor function, preserving mitochondrial function, and increasing NO· bioavailability [91].

### 5.3. Effects of H_2_ on Nervous System Diseases

H_2_ is engaged in the restoration of neurodegenerative diseases [92,93]. Research in our laboratory administered HRW to Alzheimer’s disease (AD) mice for 3 consecutive months to study its effect on cognitive function. The result showed that HRW significantly improved cognitive behaviors and ameliorated oxidative stress and inflammatory responses in the brains of female AD mice. Moreover, estrogen levels are closely related to mitochondrial function, e.g., 17β-estradiol enhances mitochondrial signaling clusters. Our results suggest that the effects of molecular H_2_ in female AD mice were most likely attributable to estrogen ERβ signaling [94].

Chen et al. reported that H_2_ treatment blocks the opening of the mitochondrial permeability transition pore in neurons. Inhalation of 75% H_2_ ameliorates mechanical damage to spinal cord neurons in a dose-dependent manner, significantly inhibits the production of ROS and oxidative stress markers, inhibits neuronal apoptosis, and restores mitochondrial function [25].

The results of a clinical trial on Parkinson’s disease showed that H_2_ significantly improved neurodegenerative symptoms with a therapeutic effect comparable to non-ergot dopamine treatment. Researchers hypothesized that this may be achieved by H_2_ improving cellular energy metabolism by targeting mitochondria [95]. In another experiment, H_2_ treatment significantly increased the levels of ATP and Δψm in neuroblastoma [57], further confirming the role of H_2_ in activating oxidative phospho-rylation and mitochondrial energy.

HRS improves neuronal ischemia-reperfusion by improving mitochondrial function and reducing oxidative stress [96]. Earlier studies found that H_2_ restored mitochondrial structural damage while reducing microRNA-210 in a hypoxia-reperfusion neural model [97]. HRS also ameliorated the activation of caspase-3, attenuated ROS accumulation, closed mitochondrial permeability transition pores, and restored mitochondrial membrane potential in isoflurane-induced, cognitively impaired mice. This suggests that HRS has the potential to attenuate anesthetic neurotoxicity [98].

### 5.4. Effects of H_2_ on Digestive System Diseases

The majority of gastrointestinal microbial species show a genetic ability to metabolize H_2_, which means that H_2_ may influence the composition of gut bacteria and modulate digestive-related diseases [99,100]. It was found that HW inhibited rat intestinal I/R-induced oxidative stress, apoptosis, and inflammation [101].

Clinical data suggest that H_2_ may improve glucose metabolism by interfering with the gut microbiota of impaired fasting glucose patients [102]. Another study in patients with clinical stage IV colorectal cancer found that H_2_ inhalation activated PGC-1α expression and enhanced mitochondrial activity, thereby reducing the proportion of PD-1 and CD8^+^ T cells. The reduction of these cells was associated with improved cancer prognosis [103].

### 5.5. Effects of H_2_ on Metabolic Syndrome

Mitochondrial dysfunction results in reduced mitochondrial biogenesis and increased ROS, which has been involved in the pathogenesis of a number of metabolic diseases, including diabetes and obesity. It has been widely demonstrated that H_2_ can scavenge ROS directly by inhibiting ROS production or indirectly by enhancing antioxidant enzyme activity, suggesting that this may be contributing to the improved mitochondrial function in metabolic disorders. Numerous studies have proven the protective effects of H_2_ on metabolic syndrome, which include lowering total cholesterol, total triglycerides (TG), and low-density lipoprotein (LDL) [104], reducing serum glucose and insulin levels in mice [105], as well as modifying adiposity and body weight in db/db obese mice [106]. The protective effect of H_2_ on diabetes and its complications may be associated with the inhibition of oxidative stress, inflammation, apoptosis, activation of the mitochondrial ATP-sensitive potassium (Mito-K-ATP) pathway, etc. [107].

Ma et al. proved that H_2_ promotes fatty acid oxidation by transporting fatty acids to mitochondria and subsequent catabolism to ketone bodies in rats [108]. A clinical study evaluated the effects of H_2_ supplementation in ten middle-aged overweight women on the indicators such as hormonal status and mitochondrial function. The results showed a significant decrease in body fat, arm fat index, serum TG, and insulin levels after 4 weeks of oral administration of H_2_-generating minerals. Fasting blood lactate accumulation reflects mitochondrial dysfunction which, in turn, affects the risk of metabolic diseases. After H_2_ intervention lasting 4 weeks, blood lactate levels were significantly lower than those in the placebo group, implying that the improvement in mitochondrial function may be related to the anti-obesity effect of H_2_ [109]. However, due to the small number of subjects in this study, the reliability of this result is limited, a long-term large-scale trial is needed to further verify the improvement of H_2_ on obesity.

Another clinical study in our lab suggests that H_2_ may have a potentially beneficial effect on glucose metabolism by interfering with the gut microbiota of individuals with impaired fasting glucose. Not only that, HRW may play an important role in reducing body fat and reducing fatty liver. This suggests its potential as a therapeutic intervention to improve lipid metabolism and liver health [102].

### 5.6. The Others

H_2_ restores mitochondrial oxidoreductase activity while preventing the downward trend of mitochondrial membrane potential. It ameliorated tertbutyl hydroperoxide-induced THP-1 (human acute monocytic leukemia cell line) cytotoxicity by inhibiting fatty acid peroxidation and mitochondrial dysfunction [110].

Mikako et al. reported a 12-week double-blind trial of five patients with progressive muscular dystrophy (PMD), four patients with polymyositis/dermatomyositis (PM/DM), and five patients with mitochondrial myopathy (MM), in which the patients consumed 1.0 L of HRW per day, and 18 serum markers were measured every four weeks. The results showed a significant improvement in lactate levels in the MM patients after drinking HRW. The lactate-to-pyruvate ratio in patients with DM also showed a favorable response [28].

## 6. Conclusions and Perspectives

In conclusion, H_2_ medicine has risen as a bright star in gas medicine, but it faces a few problems. Firstly, in the H_2_ basic research area, although a large number of H_2_ medicine-related studies have been carried out, the mechanisms of H_2_ effects are quite controversial. People do not have a high level of awareness of H_2_ and doubts still exist about the efficacy and safety of H_2_. Therefore, more specific and clear mechanisms need to be clarified. This requires more outstanding scientists to join and expend greater efforts. This review attempts to challenge the view that H_2_ is a selective ^•^OH scavenger by proposing that H_2_ is a mitochondria-targeting molecule/nutrient via activating the Keap1-Nrf2 antioxidant system. Of course, this is quite a premature idea and needs more and further investigations to test and challenge.

Secondly, in the H_2_ industry, the market demand for H_2_ health products is insufficient. There are still many technical bottlenecks in the H_2_ medicine industry, such as low efficiency of H_2_ preparation and high storage and transport costs. In addition, the industrial chain of H_2_ medicine is incomplete and lacks the development of relevant standards. The H_2_ health industry involves a number of links, such as H_2_ preparation, storage, and transport; H_2_ generators; H_2_ testing; etc. At present, these links have not formed a complete industrial chain; the connection between the links is not smooth enough. Due to the lack of complete and well-defined standards, the H_2_ industry chain is difficult to regulate with high quality.

Thirdly, there is insufficient policy support for H_2_ medicine. While the H_2_ health industry has a great potential for development, the current government support for the H_2_ health industry is insufficient, and there are some deficiencies in the policy support; e.g., there is a lack of clear policy planning and support measures. Therefore, the market prospect of the H_2_ medicine industry is promising and urgently needs to be promoted.

## Figures and Tables

**Table 1:** Classification of Nrf2 inducers targeting Keap1-Nrf2.

		Mechanism	Example
Class I	Electrophilic	Cys151-dependent compounds	Bardoxolone methyl Sulforaphane, dimethyl-fumarate
Class II		Targets Cys288	15d-PGJ_2_
Class III		Reacts with any of the three sensor cysteines Cys151/Cys273/Cys288	4-HNE, NaAsO^2^, 9-nitro-octadec-9-enoic acid
Class IV		Targets cysteines Cys77/Cys434	Pubescenoside A
Class V	Non-electrophilic	Targets Cys226/Cys613/Cys622/Cys624	H_2_O_2_, cadmium chloride, zinc chloride, prostaglandin A2
Class VI		Protein–protein interaction inhibitors (PPIs)	CPUY192018

## Data Availability

The study did not report any data. Data sharing is not applicable to this article.
